# Supplementary material for: Pyruvate Dehydrogenase Kinase 1 inhibition mediated oxidative phosphorylation enhancement in cartilage promotes osteoarthritis progression
Source: BMC Musculoskelet Disord. 2023 Jul 20;24:597. doi: 10.1186/s12891-023-06585-6 (PMC10357736; doi:10.1186/s12891-023-06585-6)
Supplement: Supplementary file 1 — Additional file 1. [file 12891_2023_6585_MOESM1_ESM.zip › BP.Gsea.1653623667859/GOBP_RNA_SPLICING.html]

Details for gene set GOBP\_RNA\_SPLICING[GSEA]

|  || Dataset | 55457\_collapsed\_to\_symbols.cls.cls #OA\_versus\_normal.cls.cls #OA\_versus\_normal\_repos |
| Phenotype | cls.cls#OA\_versus\_normal\_repos |
| Upregulated in class | normal |
| GeneSet | GOBP\_RNA\_SPLICING |
| Enrichment Score (ES) | -0.3486959 |
| Normalized Enrichment Score (NES) | -1.7684575 |
| Nominal p-value | 0.0 |
| FDR q-value | 0.11977213 |
| FWER p-Value | 0.997 |
Table: GSEA Results Summary

  

Fig 1: Enrichment plot: GOBP\_RNA\_SPLICING      
 Profile of the Running ES Score & Positions of GeneSet Members on the Rank Ordered List

  

| SYMBOL | TITLE | RANK IN GENE LIST | RANK METRIC SCORE | RUNNING ES | CORE ENRICHMENT || 1 | RTCB | "RNA 2',3'-cyclic phosphate and 5'-OH ligase [Source:HGNC Symbol;Acc:HGNC:26935]" | 78 | 0.947 | 0.0019 | No |
| 2 | LSM4 | "LSM4 homolog, U6 small nuclear RNA and mRNA degradation associated [Source:HGNC Symbol;Acc:HGNC:17259]" | 174 | 0.837 | 0.0014 | No |
| 3 | FAM172A | family with sequence similarity 172 member A [Source:HGNC Symbol;Acc:HGNC:25365] | 251 | 0.785 | 0.0021 | No |
| 4 | TXNL4A | thioredoxin like 4A [Source:HGNC Symbol;Acc:HGNC:30551] | 321 | 0.736 | 0.0028 | No |
| 5 | SF3B5 | splicing factor 3b subunit 5 [Source:HGNC Symbol;Acc:HGNC:21083] | 328 | 0.734 | 0.0086 | No |
| 6 | SRPK3 | SRSF protein kinase 3 [Source:HGNC Symbol;Acc:HGNC:11402] | 329 | 0.734 | 0.0149 | No |
| 7 | PPIE | peptidylprolyl isomerase E [Source:HGNC Symbol;Acc:HGNC:9258] | 334 | 0.733 | 0.0208 | No |
| 8 | SNRNP25 | small nuclear ribonucleoprotein U11/U12 subunit 25 [Source:HGNC Symbol;Acc:HGNC:14161] | 354 | 0.723 | 0.0254 | No |
| 9 | RNF113A | ring finger protein 113A [Source:HGNC Symbol;Acc:HGNC:12974] | 355 | 0.723 | 0.0316 | No |
| 10 | LSM3 | "LSM3 homolog, U6 small nuclear RNA and mRNA degradation associated [Source:HGNC Symbol;Acc:HGNC:17874]" | 377 | 0.716 | 0.0360 | No |
| 11 | SNRPN | small nuclear ribonucleoprotein polypeptide N [Source:HGNC Symbol;Acc:HGNC:11164] | 544 | 0.644 | 0.0283 | No |
| 12 | SCNM1 | sodium channel modifier 1 [Source:HGNC Symbol;Acc:HGNC:23136] | 557 | 0.638 | 0.0328 | No |
| 13 | RBM4B | RNA binding motif protein 4B [Source:HGNC Symbol;Acc:HGNC:28842] | 681 | 0.598 | 0.0281 | No |
| 14 | ECD | ecdysoneless cell cycle regulator [Source:HGNC Symbol;Acc:HGNC:17029] | 682 | 0.598 | 0.0332 | No |
| 15 | CLP1 | cleavage factor polyribonucleotide kinase subunit 1 [Source:HGNC Symbol;Acc:HGNC:16999] | 748 | 0.582 | 0.0329 | No |
| 16 | CRNKL1 | crooked neck pre-mRNA splicing factor 1 [Source:HGNC Symbol;Acc:HGNC:15762] | 803 | 0.568 | 0.0335 | No |
| 17 | PRPF18 | pre-mRNA processing factor 18 [Source:HGNC Symbol;Acc:HGNC:17351] | 836 | 0.559 | 0.0357 | No |
| 18 | SRSF9 | serine and arginine rich splicing factor 9 [Source:HGNC Symbol;Acc:HGNC:10791] | 882 | 0.548 | 0.0368 | No |
| 19 | PPP1R8 | protein phosphatase 1 regulatory subunit 8 [Source:HGNC Symbol;Acc:HGNC:9296] | 885 | 0.547 | 0.0413 | No |
| 20 | DDX23 | DEAD-box helicase 23 [Source:HGNC Symbol;Acc:HGNC:17347] | 913 | 0.538 | 0.0437 | No |
| 21 | USP39 | ubiquitin specific peptidase 39 [Source:HGNC Symbol;Acc:HGNC:20071] | 991 | 0.519 | 0.0420 | No |
| 22 | SNRPD3 | small nuclear ribonucleoprotein D3 polypeptide [Source:HGNC Symbol;Acc:HGNC:11160] | 1046 | 0.508 | 0.0420 | No |
| 23 | SAP18 | Sin3A associated protein 18 [Source:HGNC Symbol;Acc:HGNC:10530] | 1099 | 0.496 | 0.0421 | No |
| 24 | HTATSF1 | HIV-1 Tat specific factor 1 [Source:HGNC Symbol;Acc:HGNC:5276] | 1157 | 0.485 | 0.0417 | No |
| 25 | CD2BP2 | CD2 cytoplasmic tail binding protein 2 [Source:HGNC Symbol;Acc:HGNC:1656] | 1158 | 0.485 | 0.0458 | No |
| 26 | UBL5 | ubiquitin like 5 [Source:HGNC Symbol;Acc:HGNC:13736] | 1160 | 0.485 | 0.0499 | No |
| 27 | MBNL3 | muscleblind like splicing regulator 3 [Source:HGNC Symbol;Acc:HGNC:20564] | 1165 | 0.483 | 0.0537 | No |
| 28 | SNRPD2 | small nuclear ribonucleoprotein D2 polypeptide [Source:HGNC Symbol;Acc:HGNC:11159] | 1290 | 0.460 | 0.0477 | No |
| 29 | GEMIN2 | gem nuclear organelle associated protein 2 [Source:HGNC Symbol;Acc:HGNC:10884] | 1322 | 0.454 | 0.0491 | No |
| 30 | BCAS2 | BCAS2 pre-mRNA processing factor [Source:HGNC Symbol;Acc:HGNC:975] | 1356 | 0.449 | 0.0503 | No |
| 31 | BUD31 | BUD31 homolog [Source:HGNC Symbol;Acc:HGNC:29629] | 1380 | 0.446 | 0.0523 | No |
| 32 | LSM1 | "LSM1 homolog, mRNA degradation associated [Source:HGNC Symbol;Acc:HGNC:20472]" | 1470 | 0.433 | 0.0489 | No |
| 33 | PSIP1 | PC4 and SFRS1 interacting protein 1 [Source:HGNC Symbol;Acc:HGNC:9527] | 1482 | 0.431 | 0.0517 | No |
| 34 | PPIH | peptidylprolyl isomerase H [Source:HGNC Symbol;Acc:HGNC:14651] | 1555 | 0.419 | 0.0495 | No |
| 35 | SNRPA | small nuclear ribonucleoprotein polypeptide A [Source:HGNC Symbol;Acc:HGNC:11151] | 1670 | 0.403 | 0.0439 | No |
| 36 | CLNS1A | chloride nucleotide-sensitive channel 1A [Source:HGNC Symbol;Acc:HGNC:2080] | 1671 | 0.403 | 0.0473 | No |
| 37 | RBM23 | RNA binding motif protein 23 [Source:HGNC Symbol;Acc:HGNC:20155] | 1678 | 0.402 | 0.0503 | No |
| 38 | ARL6IP4 | ADP ribosylation factor like GTPase 6 interacting protein 4 [Source:HGNC Symbol;Acc:HGNC:18076] | 1725 | 0.394 | 0.0500 | No |
| 39 | BRDT | bromodomain testis associated [Source:HGNC Symbol;Acc:HGNC:1105] | 1812 | 0.382 | 0.0464 | No |
| 40 | GPKOW | G-patch domain and KOW motifs [Source:HGNC Symbol;Acc:HGNC:30677] | 1814 | 0.382 | 0.0496 | No |
| 41 | YJU2 | YJU2 splicing factor homolog [Source:HGNC Symbol;Acc:HGNC:25518] | 1832 | 0.380 | 0.0514 | No |
| 42 | FASTK | Fas activated serine/threonine kinase [Source:HGNC Symbol;Acc:HGNC:24676] | 1834 | 0.380 | 0.0546 | No |
| 43 | SYF2 | SYF2 pre-mRNA splicing factor [Source:HGNC Symbol;Acc:HGNC:19824] | 1881 | 0.375 | 0.0541 | No |
| 44 | MFAP1 | microfibril associated protein 1 [Source:HGNC Symbol;Acc:HGNC:7032] | 1911 | 0.371 | 0.0550 | No |
| 45 | RBM4 | RNA binding motif protein 4 [Source:HGNC Symbol;Acc:HGNC:9901] | 1970 | 0.364 | 0.0535 | No |
| 46 | SRSF8 | serine and arginine rich splicing factor 8 [Source:HGNC Symbol;Acc:HGNC:16988] | 2053 | 0.356 | 0.0500 | No |
| 47 | ESRP1 | epithelial splicing regulatory protein 1 [Source:HGNC Symbol;Acc:HGNC:25966] | 2064 | 0.355 | 0.0522 | No |
| 48 | DBR1 | debranching RNA lariats 1 [Source:HGNC Symbol;Acc:HGNC:15594] | 2091 | 0.351 | 0.0531 | No |
| 49 | LSM5 | "LSM5 homolog, U6 small nuclear RNA and mRNA degradation associated [Source:HGNC Symbol;Acc:HGNC:17162]" | 2127 | 0.347 | 0.0533 | No |
| 50 | MTREX | Mtr4 exosome RNA helicase [Source:HGNC Symbol;Acc:HGNC:18734] | 2138 | 0.346 | 0.0554 | No |
| 51 | THOC7 | THO complex 7 [Source:HGNC Symbol;Acc:HGNC:29874] | 2170 | 0.341 | 0.0559 | No |
| 52 | FAM50A | family with sequence similarity 50 member A [Source:HGNC Symbol;Acc:HGNC:18786] | 2203 | 0.338 | 0.0562 | No |
| 53 | ESRP2 | epithelial splicing regulatory protein 2 [Source:HGNC Symbol;Acc:HGNC:26152] | 2435 | 0.310 | 0.0405 | No |
| 54 | DCPS | "decapping enzyme, scavenger [Source:HGNC Symbol;Acc:HGNC:29812]" | 2570 | 0.295 | 0.0323 | No |
| 55 | RBM7 | RNA binding motif protein 7 [Source:HGNC Symbol;Acc:HGNC:9904] | 2620 | 0.288 | 0.0309 | No |
| 56 | AHNAK2 | AHNAK nucleoprotein 2 [Source:HGNC Symbol;Acc:HGNC:20125] | 2738 | 0.276 | 0.0239 | No |
| 57 | RAVER2 | "ribonucleoprotein, PTB binding 2 [Source:HGNC Symbol;Acc:HGNC:25577]" | 2956 | 0.254 | 0.0088 | No |
| 58 | FXR1 | FMR1 autosomal homolog 1 [Source:HGNC Symbol;Acc:HGNC:4023] | 3012 | 0.249 | 0.0066 | No |
| 59 | C1QBP | complement C1q binding protein [Source:HGNC Symbol;Acc:HGNC:1243] | 3083 | 0.241 | 0.0031 | No |
| 60 | SF3A1 | splicing factor 3a subunit 1 [Source:HGNC Symbol;Acc:HGNC:10765] | 3152 | 0.235 | -0.0004 | No |
| 61 | GEMIN8 | gem nuclear organelle associated protein 8 [Source:HGNC Symbol;Acc:HGNC:26044] | 3156 | 0.235 | 0.0014 | No |
| 62 | PUF60 | poly(U) binding splicing factor 60 [Source:HGNC Symbol;Acc:HGNC:17042] | 3237 | 0.227 | -0.0030 | No |
| 63 | PRMT7 | protein arginine methyltransferase 7 [Source:HGNC Symbol;Acc:HGNC:25557] | 3250 | 0.226 | -0.0021 | No |
| 64 | FRG1 | FSHD region gene 1 [Source:HGNC Symbol;Acc:HGNC:3954] | 3254 | 0.226 | -0.0004 | No |
| 65 | ZMAT5 | zinc finger matrin-type 5 [Source:HGNC Symbol;Acc:HGNC:28046] | 3288 | 0.222 | -0.0011 | No |
| 66 | DDX1 | DEAD-box helicase 1 [Source:HGNC Symbol;Acc:HGNC:2734] | 3305 | 0.221 | -0.0005 | No |
| 67 | SON | SON DNA and RNA binding protein [Source:HGNC Symbol;Acc:HGNC:11183] | 3374 | 0.214 | -0.0041 | No |
| 68 | LARP7 | "La ribonucleoprotein 7, transcriptional regulator [Source:HGNC Symbol;Acc:HGNC:24912]" | 3395 | 0.212 | -0.0039 | No |
| 69 | CIRBP | cold inducible RNA binding protein [Source:HGNC Symbol;Acc:HGNC:1982] | 3441 | 0.207 | -0.0057 | No |
| 70 | SNRPF | small nuclear ribonucleoprotein polypeptide F [Source:HGNC Symbol;Acc:HGNC:11162] | 3509 | 0.201 | -0.0093 | No |
| 71 | RBFOX2 | RNA binding fox-1 homolog 2 [Source:HGNC Symbol;Acc:HGNC:9906] | 3523 | 0.200 | -0.0087 | No |
| 72 | MBNL2 | muscleblind like splicing regulator 2 [Source:HGNC Symbol;Acc:HGNC:16746] | 3524 | 0.200 | -0.0070 | No |
| 73 | RALY | RALY heterogeneous nuclear ribonucleoprotein [Source:HGNC Symbol;Acc:HGNC:15921] | 3546 | 0.198 | -0.0069 | No |
| 74 | TSEN34 | tRNA splicing endonuclease subunit 34 [Source:HGNC Symbol;Acc:HGNC:15506] | 3617 | 0.192 | -0.0109 | No |
| 75 | LSM7 | "LSM7 homolog, U6 small nuclear RNA and mRNA degradation associated [Source:HGNC Symbol;Acc:HGNC:20470]" | 3627 | 0.191 | -0.0100 | No |
| 76 | SNRPC | small nuclear ribonucleoprotein polypeptide C [Source:HGNC Symbol;Acc:HGNC:11157] | 3649 | 0.190 | -0.0100 | No |
| 77 | COIL | coilin [Source:HGNC Symbol;Acc:HGNC:2184] | 3684 | 0.187 | -0.0111 | No |
| 78 | KHDRBS3 | "KH RNA binding domain containing, signal transduction associated 3 [Source:HGNC Symbol;Acc:HGNC:18117]" | 3714 | 0.184 | -0.0119 | No |
| 79 | GEMIN6 | gem nuclear organelle associated protein 6 [Source:HGNC Symbol;Acc:HGNC:20044] | 3741 | 0.182 | -0.0124 | No |
| 80 | KHDRBS1 | "KH RNA binding domain containing, signal transduction associated 1 [Source:HGNC Symbol;Acc:HGNC:18116]" | 3748 | 0.182 | -0.0113 | No |
| 81 | RTRAF | "RNA transcription, translation and transport factor [Source:HGNC Symbol;Acc:HGNC:23169]" | 3752 | 0.181 | -0.0100 | No |
| 82 | ZRSR2 | "zinc finger CCCH-type, RNA binding motif and serine/arginine rich 2 [Source:HGNC Symbol;Acc:HGNC:23019]" | 3771 | 0.179 | -0.0099 | No |
| 83 | SNU13 | small nuclear ribonucleoprotein 13 [Source:HGNC Symbol;Acc:HGNC:7819] | 3795 | 0.178 | -0.0102 | No |
| 84 | CWF19L1 | CWF19 like cell cycle control factor 1 [Source:HGNC Symbol;Acc:HGNC:25613] | 3981 | 0.157 | -0.0236 | No |
| 85 | LSM6 | "LSM6 homolog, U6 small nuclear RNA and mRNA degradation associated [Source:HGNC Symbol;Acc:HGNC:17017]" | 4019 | 0.154 | -0.0252 | No |
| 86 | SNRNP27 | small nuclear ribonucleoprotein U4/U6.U5 subunit 27 [Source:HGNC Symbol;Acc:HGNC:30240] | 4037 | 0.152 | -0.0253 | No |
| 87 | TMBIM6 | transmembrane BAX inhibitor motif containing 6 [Source:HGNC Symbol;Acc:HGNC:11723] | 4045 | 0.152 | -0.0246 | No |
| 88 | PPP2CA | protein phosphatase 2 catalytic subunit alpha [Source:HGNC Symbol;Acc:HGNC:9299] | 4146 | 0.143 | -0.0313 | No |
| 89 | MAGOH | "mago homolog, exon junction complex subunit [Source:HGNC Symbol;Acc:HGNC:6815]" | 4266 | 0.131 | -0.0396 | No |
| 90 | ESS2 | ess-2 splicing factor homolog [Source:HGNC Symbol;Acc:HGNC:16817] | 4347 | 0.124 | -0.0449 | No |
| 91 | GPATCH1 | G-patch domain containing 1 [Source:HGNC Symbol;Acc:HGNC:24658] | 4406 | 0.120 | -0.0485 | No |
| 92 | SNRNP35 | small nuclear ribonucleoprotein U11/U12 subunit 35 [Source:HGNC Symbol;Acc:HGNC:30852] | 4547 | 0.109 | -0.0587 | No |
| 93 | KHDRBS2 | "KH RNA binding domain containing, signal transduction associated 2 [Source:HGNC Symbol;Acc:HGNC:18114]" | 4550 | 0.109 | -0.0580 | No |
| 94 | PRPF6 | pre-mRNA processing factor 6 [Source:HGNC Symbol;Acc:HGNC:15860] | 4567 | 0.108 | -0.0583 | No |
| 95 | PCBP4 | poly(rC) binding protein 4 [Source:HGNC Symbol;Acc:HGNC:8652] | 4577 | 0.107 | -0.0581 | No |
| 96 | LSM2 | "LSM2 homolog, U6 small nuclear RNA and mRNA degradation associated [Source:HGNC Symbol;Acc:HGNC:13940]" | 4641 | 0.101 | -0.0623 | No |
| 97 | PTBP1 | polypyrimidine tract binding protein 1 [Source:HGNC Symbol;Acc:HGNC:9583] | 4650 | 0.100 | -0.0621 | No |
| 98 | GRSF1 | G-rich RNA sequence binding factor 1 [Source:HGNC Symbol;Acc:HGNC:4610] | 4660 | 0.099 | -0.0619 | No |
| 99 | DDX47 | DEAD-box helicase 47 [Source:HGNC Symbol;Acc:HGNC:18682] | 4789 | 0.088 | -0.0714 | No |
| 100 | DDX41 | DEAD-box helicase 41 [Source:HGNC Symbol;Acc:HGNC:18674] | 5004 | 0.073 | -0.0878 | No |
| 101 | NRDE2 | "NRDE-2, necessary for RNA interference, domain containing [Source:HGNC Symbol;Acc:HGNC:20186]" | 5019 | 0.071 | -0.0883 | No |
| 102 | PRPF4 | pre-mRNA processing factor 4 [Source:HGNC Symbol;Acc:HGNC:17349] | 5076 | 0.067 | -0.0922 | No |
| 103 | IK | IK cytokine [Source:HGNC Symbol;Acc:HGNC:5958] | 5225 | 0.054 | -0.1035 | No |
| 104 | CDC40 | cell division cycle 40 [Source:HGNC Symbol;Acc:HGNC:17350] | 5228 | 0.054 | -0.1032 | No |
| 105 | SNRPB2 | small nuclear ribonucleoprotein polypeptide B2 [Source:HGNC Symbol;Acc:HGNC:11155] | 5266 | 0.050 | -0.1057 | No |
| 106 | PRPF8 | pre-mRNA processing factor 8 [Source:HGNC Symbol;Acc:HGNC:17340] | 5287 | 0.049 | -0.1069 | No |
| 107 | RPS26 | ribosomal protein S26 [Source:HGNC Symbol;Acc:HGNC:10414] | 5368 | 0.043 | -0.1129 | No |
| 108 | STRAP | serine/threonine kinase receptor associated protein [Source:HGNC Symbol;Acc:HGNC:30796] | 5375 | 0.042 | -0.1130 | No |
| 109 | WT1 | WT1 transcription factor [Source:HGNC Symbol;Acc:HGNC:12796] | 5452 | 0.037 | -0.1187 | No |
| 110 | CDC5L | cell division cycle 5 like [Source:HGNC Symbol;Acc:HGNC:1743] | 5460 | 0.036 | -0.1190 | No |
| 111 | SNW1 | SNW domain containing 1 [Source:HGNC Symbol;Acc:HGNC:16696] | 5472 | 0.035 | -0.1195 | No |
| 112 | DHX8 | DEAH-box helicase 8 [Source:HGNC Symbol;Acc:HGNC:2749] | 5531 | 0.030 | -0.1239 | No |
| 113 | SNRPE | small nuclear ribonucleoprotein polypeptide E [Source:HGNC Symbol;Acc:HGNC:11161] | 5551 | 0.029 | -0.1252 | No |
| 114 | ERN1 | endoplasmic reticulum to nucleus signaling 1 [Source:HGNC Symbol;Acc:HGNC:3449] | 5658 | 0.019 | -0.1334 | No |
| 115 | SART1 | "spliceosome associated factor 1, recruiter of U4/U6.U5 tri-snRNP [Source:HGNC Symbol;Acc:HGNC:10538]" | 5690 | 0.017 | -0.1358 | No |
| 116 | DDX46 | DEAD-box helicase 46 [Source:HGNC Symbol;Acc:HGNC:18681] | 5744 | 0.012 | -0.1399 | No |
| 117 | HNRNPH3 | heterogeneous nuclear ribonucleoprotein H3 [Source:HGNC Symbol;Acc:HGNC:5043] | 5745 | 0.012 | -0.1398 | No |
| 118 | USB1 | U6 snRNA biogenesis phosphodiesterase 1 [Source:HGNC Symbol;Acc:HGNC:25792] | 5797 | 0.008 | -0.1438 | No |
| 119 | PQBP1 | polyglutamine binding protein 1 [Source:HGNC Symbol;Acc:HGNC:9330] | 5827 | 0.007 | -0.1460 | No |
| 120 | SF3B2 | splicing factor 3b subunit 2 [Source:HGNC Symbol;Acc:HGNC:10769] | 5895 | 0.002 | -0.1513 | No |
| 121 | SF3A2 | splicing factor 3a subunit 2 [Source:HGNC Symbol;Acc:HGNC:10766] | 5999 | -0.004 | -0.1595 | No |
| 122 | RBMXL2 | RBMX like 2 [Source:HGNC Symbol;Acc:HGNC:17886] | 6034 | -0.007 | -0.1621 | No |
| 123 | RBM42 | RNA binding motif protein 42 [Source:HGNC Symbol;Acc:HGNC:28117] | 6059 | -0.009 | -0.1640 | No |
| 124 | MPHOSPH10 | M-phase phosphoprotein 10 [Source:HGNC Symbol;Acc:HGNC:7213] | 6088 | -0.011 | -0.1661 | No |
| 125 | RBFOX1 | RNA binding fox-1 homolog 1 [Source:HGNC Symbol;Acc:HGNC:18222] | 6152 | -0.016 | -0.1710 | No |
| 126 | RRAGC | Ras related GTP binding C [Source:HGNC Symbol;Acc:HGNC:19902] | 6202 | -0.021 | -0.1747 | No |
| 127 | PRMT1 | protein arginine methyltransferase 1 [Source:HGNC Symbol;Acc:HGNC:5187] | 6319 | -0.029 | -0.1837 | No |
| 128 | TSEN2 | tRNA splicing endonuclease subunit 2 [Source:HGNC Symbol;Acc:HGNC:28422] | 6331 | -0.031 | -0.1843 | No |
| 129 | SF3B3 | splicing factor 3b subunit 3 [Source:HGNC Symbol;Acc:HGNC:10770] | 6372 | -0.033 | -0.1872 | No |
| 130 | AAR2 | AAR2 splicing factor [Source:HGNC Symbol;Acc:HGNC:15886] | 6401 | -0.035 | -0.1891 | No |
| 131 | CLK3 | CDC like kinase 3 [Source:HGNC Symbol;Acc:HGNC:2071] | 6417 | -0.037 | -0.1900 | No |
| 132 | SNIP1 | Smad nuclear interacting protein 1 [Source:HGNC Symbol;Acc:HGNC:30587] | 6466 | -0.040 | -0.1935 | No |
| 133 | WTAP | WT1 associated protein [Source:HGNC Symbol;Acc:HGNC:16846] | 6598 | -0.049 | -0.2035 | No |
| 134 | RBM10 | RNA binding motif protein 10 [Source:HGNC Symbol;Acc:HGNC:9896] | 6647 | -0.053 | -0.2068 | No |
| 135 | WBP4 | WW domain binding protein 4 [Source:HGNC Symbol;Acc:HGNC:12739] | 6678 | -0.054 | -0.2088 | No |
| 136 | METTL3 | "methyltransferase 3, N6-adenosine-methyltransferase complex catalytic subunit [Source:HGNC Symbol;Acc:HGNC:17563]" | 6685 | -0.055 | -0.2088 | No |
| 137 | SF3B4 | splicing factor 3b subunit 4 [Source:HGNC Symbol;Acc:HGNC:10771] | 6777 | -0.061 | -0.2155 | No |
| 138 | PRDX6 | peroxiredoxin 6 [Source:HGNC Symbol;Acc:HGNC:16753] | 6783 | -0.061 | -0.2154 | No |
| 139 | LSM8 | "LSM8 homolog, U6 small nuclear RNA associated [Source:HGNC Symbol;Acc:HGNC:20471]" | 6828 | -0.065 | -0.2183 | No |
| 140 | RBM15B | RNA binding motif protein 15B [Source:HGNC Symbol;Acc:HGNC:24303] | 6867 | -0.069 | -0.2208 | No |
| 141 | HNRNPH2 | heterogeneous nuclear ribonucleoprotein H2 [Source:HGNC Symbol;Acc:HGNC:5042] | 7007 | -0.080 | -0.2311 | No |
| 142 | ZNF638 | zinc finger protein 638 [Source:HGNC Symbol;Acc:HGNC:17894] | 7038 | -0.083 | -0.2328 | No |
| 143 | POLR2A | RNA polymerase II subunit A [Source:HGNC Symbol;Acc:HGNC:9187] | 7103 | -0.088 | -0.2371 | No |
| 144 | SNRNP200 | small nuclear ribonucleoprotein U5 subunit 200 [Source:HGNC Symbol;Acc:HGNC:30859] | 7249 | -0.099 | -0.2478 | No |
| 145 | SUGP1 | SURP and G-patch domain containing 1 [Source:HGNC Symbol;Acc:HGNC:18643] | 7267 | -0.100 | -0.2483 | No |
| 146 | PRKRIP1 | PRKR interacting protein 1 [Source:HGNC Symbol;Acc:HGNC:21894] | 7282 | -0.101 | -0.2486 | No |
| 147 | SMU1 | SMU1 DNA replication regulator and spliceosomal factor [Source:HGNC Symbol;Acc:HGNC:18247] | 7299 | -0.102 | -0.2490 | No |
| 148 | WBP11 | WW domain binding protein 11 [Source:HGNC Symbol;Acc:HGNC:16461] | 7312 | -0.103 | -0.2491 | No |
| 149 | NOVA1 | NOVA alternative splicing regulator 1 [Source:HGNC Symbol;Acc:HGNC:7886] | 7394 | -0.110 | -0.2546 | No |
| 150 | HNRNPC | heterogeneous nuclear ribonucleoprotein C [Source:HGNC Symbol;Acc:HGNC:5035] | 7437 | -0.114 | -0.2570 | No |
| 151 | CWC25 | CWC25 spliceosome associated protein homolog [Source:HGNC Symbol;Acc:HGNC:25989] | 7482 | -0.117 | -0.2595 | No |
| 152 | MAGOHB | "mago homolog B, exon junction complex subunit [Source:HGNC Symbol;Acc:HGNC:25504]" | 7506 | -0.119 | -0.2603 | No |
| 153 | NOL3 | nucleolar protein 3 [Source:HGNC Symbol;Acc:HGNC:7869] | 7545 | -0.122 | -0.2623 | No |
| 154 | RBMX2 | RNA binding motif protein X-linked 2 [Source:HGNC Symbol;Acc:HGNC:24282] | 7614 | -0.128 | -0.2666 | No |
| 155 | EIF4A3 | eukaryotic translation initiation factor 4A3 [Source:HGNC Symbol;Acc:HGNC:18683] | 7646 | -0.130 | -0.2679 | No |
| 156 | AQR | aquarius intron-binding spliceosomal factor [Source:HGNC Symbol;Acc:HGNC:29513] | 7654 | -0.131 | -0.2674 | No |
| 157 | DYRK1A | dual specificity tyrosine phosphorylation regulated kinase 1A [Source:HGNC Symbol;Acc:HGNC:3091] | 7667 | -0.132 | -0.2672 | No |
| 158 | TTF2 | transcription termination factor 2 [Source:HGNC Symbol;Acc:HGNC:12398] | 7764 | -0.139 | -0.2737 | No |
| 159 | TGS1 | trimethylguanosine synthase 1 [Source:HGNC Symbol;Acc:HGNC:17843] | 7791 | -0.141 | -0.2745 | No |
| 160 | MYOD1 | myogenic differentiation 1 [Source:HGNC Symbol;Acc:HGNC:7611] | 7839 | -0.143 | -0.2771 | No |
| 161 | TARDBP | TAR DNA binding protein [Source:HGNC Symbol;Acc:HGNC:11571] | 7864 | -0.146 | -0.2777 | No |
| 162 | HSPA1A | heat shock protein family A (Hsp70) member 1A [Source:HGNC Symbol;Acc:HGNC:5232] | 7876 | -0.146 | -0.2774 | No |
| 163 | PRPF31 | pre-mRNA processing factor 31 [Source:HGNC Symbol;Acc:HGNC:15446] | 7898 | -0.147 | -0.2778 | No |
| 164 | PRMT5 | protein arginine methyltransferase 5 [Source:HGNC Symbol;Acc:HGNC:10894] | 7904 | -0.148 | -0.2769 | No |
| 165 | FMR1 | FMRP translational regulator 1 [Source:HGNC Symbol;Acc:HGNC:3775] | 7944 | -0.151 | -0.2787 | No |
| 166 | RBM12B | RNA binding motif protein 12B [Source:HGNC Symbol;Acc:HGNC:32310] | 8036 | -0.158 | -0.2846 | No |
| 167 | HNRNPM | heterogeneous nuclear ribonucleoprotein M [Source:HGNC Symbol;Acc:HGNC:5046] | 8127 | -0.165 | -0.2904 | No |
| 168 | RNPS1 | RNA binding protein with serine rich domain 1 [Source:HGNC Symbol;Acc:HGNC:10080] | 8275 | -0.177 | -0.3006 | No |
| 169 | SETX | senataxin [Source:HGNC Symbol;Acc:HGNC:445] | 8398 | -0.185 | -0.3087 | No |
| 170 | NCBP2 | nuclear cap binding protein subunit 2 [Source:HGNC Symbol;Acc:HGNC:7659] | 8478 | -0.192 | -0.3133 | No |
| 171 | THOC5 | THO complex 5 [Source:HGNC Symbol;Acc:HGNC:19074] | 8549 | -0.198 | -0.3172 | No |
| 172 | HNRNPA2B1 | heterogeneous nuclear ribonucleoprotein A2/B1 [Source:HGNC Symbol;Acc:HGNC:5033] | 8576 | -0.200 | -0.3176 | No |
| 173 | HNRNPA3 | heterogeneous nuclear ribonucleoprotein A3 [Source:HGNC Symbol;Acc:HGNC:24941] | 8603 | -0.201 | -0.3179 | No |
| 174 | KDM1A | lysine demethylase 1A [Source:HGNC Symbol;Acc:HGNC:29079] | 8608 | -0.202 | -0.3165 | No |
| 175 | RBM8A | RNA binding motif protein 8A [Source:HGNC Symbol;Acc:HGNC:9905] | 8662 | -0.205 | -0.3190 | No |
| 176 | RBM41 | RNA binding motif protein 41 [Source:HGNC Symbol;Acc:HGNC:25617] | 8705 | -0.210 | -0.3206 | No |
| 177 | SUPT6H | "SPT6 homolog, histone chaperone and transcription elongation factor [Source:HGNC Symbol;Acc:HGNC:11470]" | 8752 | -0.213 | -0.3224 | No |
| 178 | CLK4 | CDC like kinase 4 [Source:HGNC Symbol;Acc:HGNC:13659] | 8760 | -0.213 | -0.3212 | No |
| 179 | XAB2 | XPA binding protein 2 [Source:HGNC Symbol;Acc:HGNC:14089] | 8778 | -0.215 | -0.3207 | No |
| 180 | RBM3 | RNA binding motif protein 3 [Source:HGNC Symbol;Acc:HGNC:9900] | 8859 | -0.222 | -0.3252 | No |
| 181 | AFF2 | AF4/FMR2 family member 2 [Source:HGNC Symbol;Acc:HGNC:3776] | 8869 | -0.222 | -0.3240 | No |
| 182 | HNRNPL | heterogeneous nuclear ribonucleoprotein L [Source:HGNC Symbol;Acc:HGNC:5045] | 8892 | -0.224 | -0.3238 | No |
| 183 | RRP1B | ribosomal RNA processing 1B [Source:HGNC Symbol;Acc:HGNC:23818] | 8909 | -0.225 | -0.3232 | No |
| 184 | RBMX | RNA binding motif protein X-linked [Source:HGNC Symbol;Acc:HGNC:9910] | 8938 | -0.227 | -0.3235 | No |
| 185 | RBMY1A1 | RNA binding motif protein Y-linked family 1 member A1 [Source:HGNC Symbol;Acc:HGNC:9912] | 9014 | -0.233 | -0.3275 | No |
| 186 | SNRPG | small nuclear ribonucleoprotein polypeptide G [Source:HGNC Symbol;Acc:HGNC:11163] | 9067 | -0.237 | -0.3296 | No |
| 187 | PPWD1 | peptidylprolyl isomerase domain and WD repeat containing 1 [Source:HGNC Symbol;Acc:HGNC:28954] | 9069 | -0.237 | -0.3277 | No |
| 188 | SNRPB | small nuclear ribonucleoprotein polypeptides B and B1 [Source:HGNC Symbol;Acc:HGNC:11153] | 9085 | -0.239 | -0.3268 | No |
| 189 | TFIP11 | tuftelin interacting protein 11 [Source:HGNC Symbol;Acc:HGNC:17165] | 9098 | -0.239 | -0.3257 | No |
| 190 | USP4 | ubiquitin specific peptidase 4 [Source:HGNC Symbol;Acc:HGNC:12627] | 9130 | -0.242 | -0.3261 | No |
| 191 | HSPA8 | heat shock protein family A (Hsp70) member 8 [Source:HGNC Symbol;Acc:HGNC:5241] | 9140 | -0.243 | -0.3248 | No |
| 192 | NCBP1 | nuclear cap binding protein subunit 1 [Source:HGNC Symbol;Acc:HGNC:7658] | 9149 | -0.244 | -0.3233 | No |
| 193 | ZPR1 | ZPR1 zinc finger [Source:HGNC Symbol;Acc:HGNC:13051] | 9151 | -0.244 | -0.3213 | No |
| 194 | PRPF4B | pre-mRNA processing factor 4B [Source:HGNC Symbol;Acc:HGNC:17346] | 9210 | -0.249 | -0.3238 | No |
| 195 | CDK12 | cyclin dependent kinase 12 [Source:HGNC Symbol;Acc:HGNC:24224] | 9225 | -0.250 | -0.3228 | No |
| 196 | TRA2B | transformer 2 beta homolog [Source:HGNC Symbol;Acc:HGNC:10781] | 9243 | -0.251 | -0.3220 | No |
| 197 | SNRNP40 | small nuclear ribonucleoprotein U5 subunit 40 [Source:HGNC Symbol;Acc:HGNC:30857] | 9295 | -0.256 | -0.3239 | No |
| 198 | PAXBP1 | PAX3 and PAX7 binding protein 1 [Source:HGNC Symbol;Acc:HGNC:13579] | 9333 | -0.258 | -0.3247 | No |
| 199 | NONO | non-POU domain containing octamer binding [Source:HGNC Symbol;Acc:HGNC:7871] | 9334 | -0.258 | -0.3225 | No |
| 200 | YJU2B | YJU2 splicing factor homolog B [Source:HGNC Symbol;Acc:HGNC:28118] | 9429 | -0.267 | -0.3277 | No |
| 201 | HNRNPR | heterogeneous nuclear ribonucleoprotein R [Source:HGNC Symbol;Acc:HGNC:5047] | 9430 | -0.267 | -0.3254 | No |
| 202 | GEMIN7 | gem nuclear organelle associated protein 7 [Source:HGNC Symbol;Acc:HGNC:20045] | 9441 | -0.268 | -0.3239 | No |
| 203 | LGALS3 | galectin 3 [Source:HGNC Symbol;Acc:HGNC:6563] | 9508 | -0.274 | -0.3268 | No |
| 204 | ZC3H13 | zinc finger CCCH-type containing 13 [Source:HGNC Symbol;Acc:HGNC:20368] | 9558 | -0.279 | -0.3283 | No |
| 205 | SNRPD1 | small nuclear ribonucleoprotein D1 polypeptide [Source:HGNC Symbol;Acc:HGNC:11158] | 9579 | -0.281 | -0.3275 | No |
| 206 | YBX1 | Y-box binding protein 1 [Source:HGNC Symbol;Acc:HGNC:8014] | 9623 | -0.285 | -0.3285 | No |
| 207 | ZCCHC8 | zinc finger CCHC-type containing 8 [Source:HGNC Symbol;Acc:HGNC:25265] | 9624 | -0.285 | -0.3261 | No |
| 208 | SMNDC1 | survival motor neuron domain containing 1 [Source:HGNC Symbol;Acc:HGNC:16900] | 9638 | -0.287 | -0.3247 | No |
| 209 | CIR1 | "corepressor interacting with RBPJ, CIR1 [Source:HGNC Symbol;Acc:HGNC:24217]" | 9640 | -0.287 | -0.3223 | No |
| 210 | RBM14 | RNA binding motif protein 14 [Source:HGNC Symbol;Acc:HGNC:14219] | 9663 | -0.288 | -0.3216 | No |
| 211 | SFPQ | splicing factor proline and glutamine rich [Source:HGNC Symbol;Acc:HGNC:10774] | 9747 | -0.296 | -0.3257 | No |
| 212 | RBM12 | RNA binding motif protein 12 [Source:HGNC Symbol;Acc:HGNC:9898] | 9841 | -0.305 | -0.3305 | No |
| 213 | HNRNPU | heterogeneous nuclear ribonucleoprotein U [Source:HGNC Symbol;Acc:HGNC:5048] | 9847 | -0.306 | -0.3283 | No |
| 214 | GCFC2 | GC-rich sequence DNA-binding factor 2 [Source:HGNC Symbol;Acc:HGNC:1317] | 9908 | -0.312 | -0.3304 | No |
| 215 | KHDC4 | "KH domain containing 4, pre-mRNA splicing factor [Source:HGNC Symbol;Acc:HGNC:29145]" | 9997 | -0.321 | -0.3347 | No |
| 216 | GEMIN4 | gem nuclear organelle associated protein 4 [Source:HGNC Symbol;Acc:HGNC:15717] | 10075 | -0.328 | -0.3380 | No |
| 217 | C9orf78 | chromosome 9 open reading frame 78 [Source:HGNC Symbol;Acc:HGNC:24932] | 10210 | -0.342 | -0.3458 | Yes |
| 218 | DHX35 | DEAH-box helicase 35 [Source:HGNC Symbol;Acc:HGNC:15861] | 10215 | -0.342 | -0.3432 | Yes |
| 219 | PTBP3 | polypyrimidine tract binding protein 3 [Source:HGNC Symbol;Acc:HGNC:10253] | 10259 | -0.347 | -0.3437 | Yes |
| 220 | SRSF1 | serine and arginine rich splicing factor 1 [Source:HGNC Symbol;Acc:HGNC:10780] | 10272 | -0.348 | -0.3417 | Yes |
| 221 | SRSF3 | serine and arginine rich splicing factor 3 [Source:HGNC Symbol;Acc:HGNC:10785] | 10280 | -0.349 | -0.3392 | Yes |
| 222 | SRSF5 | serine and arginine rich splicing factor 5 [Source:HGNC Symbol;Acc:HGNC:10787] | 10282 | -0.349 | -0.3363 | Yes |
| 223 | SF3A3 | splicing factor 3a subunit 3 [Source:HGNC Symbol;Acc:HGNC:10767] | 10311 | -0.352 | -0.3356 | Yes |
| 224 | LUC7L2 | "LUC7 like 2, pre-mRNA splicing factor [Source:HGNC Symbol;Acc:HGNC:21608]" | 10334 | -0.354 | -0.3343 | Yes |
| 225 | RBM22 | RNA binding motif protein 22 [Source:HGNC Symbol;Acc:HGNC:25503] | 10345 | -0.355 | -0.3321 | Yes |
| 226 | SCAF11 | SR-related CTD associated factor 11 [Source:HGNC Symbol;Acc:HGNC:10784] | 10375 | -0.358 | -0.3313 | Yes |
| 227 | PPP2R1A | protein phosphatase 2 scaffold subunit Aalpha [Source:HGNC Symbol;Acc:HGNC:9302] | 10395 | -0.360 | -0.3298 | Yes |
| 228 | ELAVL4 | ELAV like RNA binding protein 4 [Source:HGNC Symbol;Acc:HGNC:3315] | 10412 | -0.362 | -0.3280 | Yes |
| 229 | HABP4 | hyaluronan binding protein 4 [Source:HGNC Symbol;Acc:HGNC:17062] | 10485 | -0.371 | -0.3306 | Yes |
| 230 | DHX15 | DEAH-box helicase 15 [Source:HGNC Symbol;Acc:HGNC:2738] | 10498 | -0.372 | -0.3283 | Yes |
| 231 | RBM15 | RNA binding motif protein 15 [Source:HGNC Symbol;Acc:HGNC:14959] | 10499 | -0.373 | -0.3252 | Yes |
| 232 | PPARGC1A | PPARG coactivator 1 alpha [Source:HGNC Symbol;Acc:HGNC:9237] | 10504 | -0.374 | -0.3223 | Yes |
| 233 | SRSF2 | serine and arginine rich splicing factor 2 [Source:HGNC Symbol;Acc:HGNC:10783] | 10507 | -0.374 | -0.3193 | Yes |
| 234 | SRPK2 | SRSF protein kinase 2 [Source:HGNC Symbol;Acc:HGNC:11306] | 10522 | -0.376 | -0.3172 | Yes |
| 235 | MBNL1 | muscleblind like splicing regulator 1 [Source:HGNC Symbol;Acc:HGNC:6923] | 10557 | -0.379 | -0.3167 | Yes |
| 236 | DHX9 | DExH-box helicase 9 [Source:HGNC Symbol;Acc:HGNC:2750] | 10627 | -0.386 | -0.3189 | Yes |
| 237 | LUC7L3 | LUC7 like 3 pre-mRNA splicing factor [Source:HGNC Symbol;Acc:HGNC:24309] | 10647 | -0.388 | -0.3171 | Yes |
| 238 | SRSF6 | serine and arginine rich splicing factor 6 [Source:HGNC Symbol;Acc:HGNC:10788] | 10659 | -0.389 | -0.3147 | Yes |
| 239 | CDK13 | cyclin dependent kinase 13 [Source:HGNC Symbol;Acc:HGNC:1733] | 10675 | -0.390 | -0.3125 | Yes |
| 240 | YTHDC1 | YTH domain containing 1 [Source:HGNC Symbol;Acc:HGNC:30626] | 10687 | -0.391 | -0.3101 | Yes |
| 241 | U2AF1 | U2 small nuclear RNA auxiliary factor 1 [Source:HGNC Symbol;Acc:HGNC:12453] | 10762 | -0.400 | -0.3126 | Yes |
| 242 | RBM25 | RNA binding motif protein 25 [Source:HGNC Symbol;Acc:HGNC:23244] | 10800 | -0.405 | -0.3120 | Yes |
| 243 | CELF2 | CUGBP Elav-like family member 2 [Source:HGNC Symbol;Acc:HGNC:2550] | 10832 | -0.409 | -0.3110 | Yes |
| 244 | RBM5 | RNA binding motif protein 5 [Source:HGNC Symbol;Acc:HGNC:9902] | 10849 | -0.410 | -0.3088 | Yes |
| 245 | TIA1 | TIA1 cytotoxic granule associated RNA binding protein [Source:HGNC Symbol;Acc:HGNC:11802] | 10855 | -0.411 | -0.3057 | Yes |
| 246 | NUP98 | nucleoporin 98 and 96 precursor [Source:HGNC Symbol;Acc:HGNC:8068] | 10875 | -0.413 | -0.3037 | Yes |
| 247 | AHNAK | AHNAK nucleoprotein [Source:HGNC Symbol;Acc:HGNC:347] | 10903 | -0.417 | -0.3023 | Yes |
| 248 | SRSF10 | serine and arginine rich splicing factor 10 [Source:HGNC Symbol;Acc:HGNC:16713] | 10926 | -0.422 | -0.3005 | Yes |
| 249 | SRSF7 | serine and arginine rich splicing factor 7 [Source:HGNC Symbol;Acc:HGNC:10789] | 10930 | -0.422 | -0.2971 | Yes |
| 250 | SF3B1 | splicing factor 3b subunit 1 [Source:HGNC Symbol;Acc:HGNC:10768] | 10948 | -0.424 | -0.2949 | Yes |
| 251 | RBM19 | RNA binding motif protein 19 [Source:HGNC Symbol;Acc:HGNC:29098] | 11030 | -0.434 | -0.2976 | Yes |
| 252 | PIK3R1 | phosphoinositide-3-kinase regulatory subunit 1 [Source:HGNC Symbol;Acc:HGNC:8979] | 11099 | -0.443 | -0.2992 | Yes |
| 253 | PRPF38B | pre-mRNA processing factor 38B [Source:HGNC Symbol;Acc:HGNC:25512] | 11147 | -0.450 | -0.2991 | Yes |
| 254 | SYNCRIP | synaptotagmin binding cytoplasmic RNA interacting protein [Source:HGNC Symbol;Acc:HGNC:16918] | 11182 | -0.454 | -0.2980 | Yes |
| 255 | SNRPA1 | small nuclear ribonucleoprotein polypeptide A' [Source:HGNC Symbol;Acc:HGNC:11152] | 11218 | -0.459 | -0.2969 | Yes |
| 256 | PRPF19 | pre-mRNA processing factor 19 [Source:HGNC Symbol;Acc:HGNC:17896] | 11269 | -0.467 | -0.2969 | Yes |
| 257 | KHSRP | KH-type splicing regulatory protein [Source:HGNC Symbol;Acc:HGNC:6316] | 11285 | -0.469 | -0.2941 | Yes |
| 258 | SRRM1 | serine and arginine repetitive matrix 1 [Source:HGNC Symbol;Acc:HGNC:16638] | 11302 | -0.471 | -0.2913 | Yes |
| 259 | HNRNPF | heterogeneous nuclear ribonucleoprotein F [Source:HGNC Symbol;Acc:HGNC:5039] | 11339 | -0.476 | -0.2901 | Yes |
| 260 | METTL16 | "methyltransferase 16, N6-methyladenosine [Source:HGNC Symbol;Acc:HGNC:28484]" | 11346 | -0.478 | -0.2865 | Yes |
| 261 | DDX39A | DExD-box helicase 39A [Source:HGNC Symbol;Acc:HGNC:17821] | 11359 | -0.480 | -0.2834 | Yes |
| 262 | CLK2 | CDC like kinase 2 [Source:HGNC Symbol;Acc:HGNC:2069] | 11368 | -0.482 | -0.2799 | Yes |
| 263 | TXNL4B | thioredoxin like 4B [Source:HGNC Symbol;Acc:HGNC:26041] | 11370 | -0.482 | -0.2759 | Yes |
| 264 | RBM39 | RNA binding motif protein 39 [Source:HGNC Symbol;Acc:HGNC:15923] | 11434 | -0.493 | -0.2767 | Yes |
| 265 | PRX | periaxin [Source:HGNC Symbol;Acc:HGNC:13797] | 11438 | -0.494 | -0.2728 | Yes |
| 266 | IVNS1ABP | influenza virus NS1A binding protein [Source:HGNC Symbol;Acc:HGNC:16951] | 11505 | -0.501 | -0.2738 | Yes |
| 267 | CELF3 | CUGBP Elav-like family member 3 [Source:HGNC Symbol;Acc:HGNC:11967] | 11510 | -0.502 | -0.2698 | Yes |
| 268 | ZBTB7A | zinc finger and BTB domain containing 7A [Source:HGNC Symbol;Acc:HGNC:18078] | 11600 | -0.516 | -0.2725 | Yes |
| 269 | LUC7L | LUC7 like [Source:HGNC Symbol;Acc:HGNC:6723] | 11627 | -0.521 | -0.2701 | Yes |
| 270 | CASC3 | CASC3 exon junction complex subunit [Source:HGNC Symbol;Acc:HGNC:17040] | 11630 | -0.522 | -0.2658 | Yes |
| 271 | SF1 | splicing factor 1 [Source:HGNC Symbol;Acc:HGNC:12950] | 11664 | -0.527 | -0.2640 | Yes |
| 272 | DDX17 | DEAD-box helicase 17 [Source:HGNC Symbol;Acc:HGNC:2740] | 11716 | -0.535 | -0.2635 | Yes |
| 273 | SLC38A2 | solute carrier family 38 member 2 [Source:HGNC Symbol;Acc:HGNC:13448] | 11722 | -0.536 | -0.2593 | Yes |
| 274 | PRPF39 | pre-mRNA processing factor 39 [Source:HGNC Symbol;Acc:HGNC:20314] | 11854 | -0.560 | -0.2650 | Yes |
| 275 | DHX38 | DEAH-box helicase 38 [Source:HGNC Symbol;Acc:HGNC:17211] | 11860 | -0.562 | -0.2606 | Yes |
| 276 | SRPK1 | SRSF protein kinase 1 [Source:HGNC Symbol;Acc:HGNC:11305] | 11889 | -0.569 | -0.2580 | Yes |
| 277 | THOC6 | THO complex 6 [Source:HGNC Symbol;Acc:HGNC:28369] | 11918 | -0.576 | -0.2553 | Yes |
| 278 | TCERG1 | transcription elongation regulator 1 [Source:HGNC Symbol;Acc:HGNC:15630] | 11950 | -0.583 | -0.2528 | Yes |
| 279 | PNN | "pinin, desmosome associated protein [Source:HGNC Symbol;Acc:HGNC:9162]" | 11954 | -0.584 | -0.2480 | Yes |
| 280 | METTL4 | "methyltransferase 4, N6-adenosine [Source:HGNC Symbol;Acc:HGNC:24726]" | 11976 | -0.589 | -0.2447 | Yes |
| 281 | NOVA2 | NOVA alternative splicing regulator 2 [Source:HGNC Symbol;Acc:HGNC:7887] | 12006 | -0.594 | -0.2420 | Yes |
| 282 | RBM38 | RNA binding motif protein 38 [Source:HGNC Symbol;Acc:HGNC:15818] | 12026 | -0.599 | -0.2384 | Yes |
| 283 | ACIN1 | apoptotic chromatin condensation inducer 1 [Source:HGNC Symbol;Acc:HGNC:17066] | 12059 | -0.609 | -0.2357 | Yes |
| 284 | SNRNP70 | small nuclear ribonucleoprotein U1 subunit 70 [Source:HGNC Symbol;Acc:HGNC:11150] | 12073 | -0.613 | -0.2315 | Yes |
| 285 | SMN1 | "survival of motor neuron 1, telomeric [Source:HGNC Symbol;Acc:HGNC:11117]" | 12102 | -0.621 | -0.2285 | Yes |
| 286 | SART3 | "spliceosome associated factor 3, U4/U6 recycling protein [Source:HGNC Symbol;Acc:HGNC:16860]" | 12161 | -0.633 | -0.2277 | Yes |
| 287 | RSRC1 | arginine and serine rich coiled-coil 1 [Source:HGNC Symbol;Acc:HGNC:24152] | 12165 | -0.635 | -0.2225 | Yes |
| 288 | PTBP2 | polypyrimidine tract binding protein 2 [Source:HGNC Symbol;Acc:HGNC:17662] | 12211 | -0.649 | -0.2206 | Yes |
| 289 | FXR2 | FMR1 autosomal homolog 2 [Source:HGNC Symbol;Acc:HGNC:4024] | 12219 | -0.650 | -0.2156 | Yes |
| 290 | SREK1 | splicing regulatory glutamic acid and lysine rich protein 1 [Source:HGNC Symbol;Acc:HGNC:17882] | 12221 | -0.651 | -0.2102 | Yes |
| 291 | U2AF2 | U2 small nuclear RNA auxiliary factor 2 [Source:HGNC Symbol;Acc:HGNC:23156] | 12226 | -0.652 | -0.2049 | Yes |
| 292 | NCL | nucleolin [Source:HGNC Symbol;Acc:HGNC:7667] | 12255 | -0.662 | -0.2015 | Yes |
| 293 | CACTIN | "cactin, spliceosome C complex subunit [Source:HGNC Symbol;Acc:HGNC:29938]" | 12270 | -0.668 | -0.1969 | Yes |
| 294 | SUGP2 | SURP and G-patch domain containing 2 [Source:HGNC Symbol;Acc:HGNC:18641] | 12284 | -0.671 | -0.1923 | Yes |
| 295 | RBM28 | RNA binding motif protein 28 [Source:HGNC Symbol;Acc:HGNC:21863] | 12315 | -0.681 | -0.1889 | Yes |
| 296 | SRSF11 | serine and arginine rich splicing factor 11 [Source:HGNC Symbol;Acc:HGNC:10782] | 12322 | -0.683 | -0.1835 | Yes |
| 297 | AKAP17A | A-kinase anchoring protein 17A [Source:HGNC Symbol;Acc:HGNC:18783] | 12327 | -0.684 | -0.1780 | Yes |
| 298 | RPS13 | ribosomal protein S13 [Source:HGNC Symbol;Acc:HGNC:10386] | 12334 | -0.687 | -0.1726 | Yes |
| 299 | QKI | "QKI, KH domain containing RNA binding [Source:HGNC Symbol;Acc:HGNC:21100]" | 12340 | -0.688 | -0.1672 | Yes |
| 300 | HNRNPK | heterogeneous nuclear ribonucleoprotein K [Source:HGNC Symbol;Acc:HGNC:5044] | 12341 | -0.688 | -0.1613 | Yes |
| 301 | CLK1 | CDC like kinase 1 [Source:HGNC Symbol;Acc:HGNC:2068] | 12354 | -0.693 | -0.1564 | Yes |
| 302 | HNRNPA1 | heterogeneous nuclear ribonucleoprotein A1 [Source:HGNC Symbol;Acc:HGNC:5031] | 12356 | -0.693 | -0.1506 | Yes |
| 303 | PPIG | peptidylprolyl isomerase G [Source:HGNC Symbol;Acc:HGNC:14650] | 12358 | -0.695 | -0.1447 | Yes |
| 304 | REST | RE1 silencing transcription factor [Source:HGNC Symbol;Acc:HGNC:9966] | 12382 | -0.703 | -0.1406 | Yes |
| 305 | CELF1 | CUGBP Elav-like family member 1 [Source:HGNC Symbol;Acc:HGNC:2549] | 12391 | -0.704 | -0.1352 | Yes |
| 306 | CLASRP | CLK4 associating serine/arginine rich protein [Source:HGNC Symbol;Acc:HGNC:17731] | 12394 | -0.705 | -0.1294 | Yes |
| 307 | DHX16 | DEAH-box helicase 16 [Source:HGNC Symbol;Acc:HGNC:2739] | 12414 | -0.712 | -0.1248 | Yes |
| 308 | CCNL2 | cyclin L2 [Source:HGNC Symbol;Acc:HGNC:20570] | 12425 | -0.716 | -0.1195 | Yes |
| 309 | THRAP3 | thyroid hormone receptor associated protein 3 [Source:HGNC Symbol;Acc:HGNC:22964] | 12426 | -0.717 | -0.1134 | Yes |
| 310 | TRA2A | transformer 2 alpha homolog [Source:HGNC Symbol;Acc:HGNC:16645] | 12479 | -0.740 | -0.1113 | Yes |
| 311 | THOC1 | THO complex 1 [Source:HGNC Symbol;Acc:HGNC:19070] | 12503 | -0.749 | -0.1067 | Yes |
| 312 | HNRNPH1 | heterogeneous nuclear ribonucleoprotein H1 [Source:HGNC Symbol;Acc:HGNC:5041] | 12554 | -0.776 | -0.1041 | Yes |
| 313 | PABPC1 | poly(A) binding protein cytoplasmic 1 [Source:HGNC Symbol;Acc:HGNC:8554] | 12562 | -0.778 | -0.0980 | Yes |
| 314 | PRPF40A | pre-mRNA processing factor 40 homolog A [Source:HGNC Symbol;Acc:HGNC:16463] | 12651 | -0.826 | -0.0980 | Yes |
| 315 | JMJD6 | "jumonji domain containing 6, arginine demethylase and lysine hydroxylase [Source:HGNC Symbol;Acc:HGNC:19355]" | 12658 | -0.832 | -0.0914 | Yes |
| 316 | AKAP8L | A-kinase anchoring protein 8 like [Source:HGNC Symbol;Acc:HGNC:29857] | 12665 | -0.836 | -0.0848 | Yes |
| 317 | SRRM2 | serine/arginine repetitive matrix 2 [Source:HGNC Symbol;Acc:HGNC:16639] | 12666 | -0.837 | -0.0776 | Yes |
| 318 | DAZAP1 | DAZ associated protein 1 [Source:HGNC Symbol;Acc:HGNC:2683] | 12686 | -0.854 | -0.0719 | Yes |
| 319 | THOC2 | THO complex 2 [Source:HGNC Symbol;Acc:HGNC:19073] | 12691 | -0.855 | -0.0649 | Yes |
| 320 | RBM6 | RNA binding motif protein 6 [Source:HGNC Symbol;Acc:HGNC:9903] | 12701 | -0.865 | -0.0583 | Yes |
| 321 | CCNL1 | cyclin L1 [Source:HGNC Symbol;Acc:HGNC:20569] | 12708 | -0.870 | -0.0513 | Yes |
| 322 | FUS | FUS RNA binding protein [Source:HGNC Symbol;Acc:HGNC:4010] | 12739 | -0.902 | -0.0461 | Yes |
| 323 | SFSWAP | splicing factor SWAP [Source:HGNC Symbol;Acc:HGNC:10790] | 12747 | -0.908 | -0.0389 | Yes |
| 324 | SRSF4 | serine and arginine rich splicing factor 4 [Source:HGNC Symbol;Acc:HGNC:10786] | 12749 | -0.911 | -0.0312 | Yes |
| 325 | DDX39B | DExD-box helicase 39B [Source:HGNC Symbol;Acc:HGNC:13917] | 12783 | -0.941 | -0.0258 | Yes |
| 326 | SREK1IP1 | SREK1 interacting protein 1 [Source:HGNC Symbol;Acc:HGNC:26716] | 12810 | -0.988 | -0.0195 | Yes |
| 327 | TAF15 | TATA-box binding protein associated factor 15 [Source:HGNC Symbol;Acc:HGNC:11547] | 12828 | -1.023 | -0.0121 | Yes |
| 328 | DDX5 | DEAD-box helicase 5 [Source:HGNC Symbol;Acc:HGNC:2746] | 12834 | -1.038 | -0.0037 | Yes |
| 329 | PRPF3 | pre-mRNA processing factor 3 [Source:HGNC Symbol;Acc:HGNC:17348] | 12837 | -1.040 | 0.0050 | Yes |
Table: GSEA details [plain text format]

  

Fig 2: GOBP\_RNA\_SPLICING      
 Blue-Pink O' Gram in the Space of the Analyzed GeneSet

  

Fig 3: GOBP\_RNA\_SPLICING: Random ES distribution      
 Gene set null distribution of ES for **GOBP\_RNA\_SPLICING**
